# Thiol-based redox sensing regulates the yellow pigment and antioxidant accumulation and improves the nutritional quality of wheat grains (*Triticum aestivum* L.)

**DOI:** 10.3389/fpls.2025.1488697

**Published:** 2025-05-30

**Authors:** Ranjeet R. Kumar, Prashant Babu H, Sumedha Hasija, Mallesh Gampa, Suneha Goswami, Vinutha T., Sudhir Kumar, Gyan P. Mishra, Dwijesh Mishra, Gyanendra K. Rai, Girish K. Jha, Soora Naresh Kumar, Shelly Praveen, Aruna Tyagi, Viswanathan C.

**Affiliations:** ^1^ Division of Biochemistry, Indian Council of Agricultural Research (ICAR)-Indian Agricultural Research Institute, New Delhi, India; ^2^ Division of Genetics, Indian Council of Agricultural Research (ICAR)-Indian Agricultural Research Institute, New Delhi, India; ^3^ Division of Plant Physiology, Indian Council of Agricultural Research (ICAR)-Indian Agricultural Research Institute, New Delhi, India; ^4^ Division of Seed Technology, Indian Council of Agricultural Research (ICAR)-Indian Agricultural Research Institute, New Delhi, India; ^5^ CABin, ICAR-Indian Agricultural Statistics Research Institute, New Delhi, India; ^6^ Sher-e-Kashmir University of Agricultural Sciences and Technology, Jammu, India; ^7^ Centre for Environment Science and Climate Resilient Agriculture (CESCRA), Indian Agricultural Research Institute, New Delhi, India

**Keywords:** thiol, redox sensor, grain quality, Trx, GRX, glutathione peroxidase, yellow pigment

## Abstract

Thiol-based redox sensing has been found to play diverse roles in regulating various metabolic pathways. Here, the thiol-based redox system of 16 diverse genotypes of wheat grains was characterized, and it correlated with the accumulation of macro-/micronutrients inside the grains. We observed significant variations in the thiol and disulfide content in the grains. An expression analysis of the genes responsible for thiol-based redox sensing, such as thioredoxin (*TRX*), glutaredoxin (*GRX*) and glutathione reductase (*GR*), showed maximum fold expression in wheat *cvs*. Halna and HD2985 (high thiol *cvs*.) during the seed hardening stage (G_2_) of endosperm, as compared to low thiol-containing *cvs*. We retrieved the amino acid sequences of 11 genes linked with nutrient biosynthesis pathways and observed the highest cysteine (Cys) (2.25%) in Granule bound starch synthase (*GBSS;* involved in starch biosynthesis) and methionine (Met) (4.04%) in the *BCH* gene (involved in tannin synthesis). Genotypes with a Cys : Met ratio >1.0 were observed to be nutrient-rich and robust due to the high stability of key proteins and enzymes. The yellow pigment (shining factor) was observed to be the highest in the grains of wheat cv. NIAW34 (6.08 µg/g dry matter) with a Cys: Met ratio of 2.15. Antioxidants such as total phenolic content and tannin were observed to be significantly higher in *cvs*. (Halna, HI1544, etc.) with a ratio of Cys: Met ≥2.0. The highest level of polysaccharides (starch and resistant starch) was observed in the grains of wheat cv. HD1914 with a Cys : Met ratio of 4.0. The results of Pearson’s correlation indicated a negative relationship between thiol content and nutrient-linked traits such as total protein, gluten, and phytic acid. Micronutrients such as iron and zinc showed a weak positive correlation with thiol content. The role of thiol-based redox sensors needs to be further explored and utilized for manipulating the tolerance level and nutrient compositions of wheat grains. This will help in developing “nutrient-smart grain” and “climate-smart” crops with improved downstream processing and dough engineering.

## Introduction

Cereals are the predominant grain crops, providing a major share of carbohydrates and calories in the diet. They have an adequate amount of nutrients required for the body, though they are poor in some of the important amino acids and micronutrients (Ciudad-Mulero et al., 2021). The bioavailability of most of the nutrients has been observed to be compromised in different cereals. Wheat is a staple grain crop utilized in a large part of the world as chapatti, bread, and many other formulations. Wheat grains are rich in carbohydrates, especially polysaccharides such as starch, and also contain protein, lipids, and other nutrients (Kumar et al., 2019). The protein content of the grains varies between 7%–22% (Wieser et al., 2023). The critical phases of grain filling, mainly the grain ripening and seed desiccation stages, decide the yield and quality of the wheat grains in terms of nutrient compositions (Zhang et al., 2021). Adverse environmental conditions during the grain-filling stage in wheat have a severe effect on the physical and chemical quality of the grains (Fernie et al., 2022). The nutrient composition of wheat flour has been reported to have wide diversity across the genotypes. The effect of agro-geo-climate conditions, fertilizers, heat, drought, salinity, and other biotic/abiotic stresses on the proximate composition of wheat is well elucidated. The regulation underlying the variations in the proximate composition has been observed to be related to different biochemical metabolites synthesizing and accumulating inside the grains (Kumar et al., 2019; Grace and Henry, 2020). The metabolic pathway-associated enzymes have been reported to show significant variation in activity with changes in the accumulation of different metabolites (Cañas et al., 2017).

Cells have an inherent potential to sense their environment and adapt themselves accordingly by regulating the networks of genes and proteins. Proteins have evolved with time and show remarkable features in sensing the environment and regulating the expression of genes and the activity of enzymes. The binding of cofactors provides more specificity to the proteins in sensing the cytoplasmic/environmental changes.

Starch is one of the predominant polysaccharides present in most cereals and millets (Kumar et al., 2019). It consists of amylose and amylopectin and determines the yield and quality of the grains. Currently, lifestyle diseases such as diabetes are more prevalent and have been reported to be due to diets rich in carbohydrates (Kumar and Pathak, 2014). Starch has different fractions present in the flour, and a few of them have been recommended for patients with diabetes (Bajka et al., 2021). Resistant starch (RS) is a fraction that is a slow-digestible starch, which takes time to be digested and releases glucose slowly into the blood upon feeding. There are different types of RS, and each has a different mode of synthesis. For example, RS1 is physically not accessible to the amylase enzyme and is present in = coarse grains; RS2 is granular starch extracted from raw potatos, bananas, etc.; RS3 is synthesized though heating and cooling of starch; RS4 is chemically modified starch; and RS5 is a amylose–lipid complex (Harris, 2019). Researchers have reported wide variations in the resistant starch content of different agriculturally important crops such as wheat, maize, rice, and millet (Zheng et al., 2020). Wheat has ~0.75% to 1.0% RS in the grains (Kumar et al., 2019). The mechanism underlying the variation in the proportion of RS is mostly enzymatically based and has not been much characterized. A high RS content in flour has been considered one of the desirable traits for good quality grains and flours. RS has been observed to enhance the texture, integrity, and quality of flour (Khan et al., 2020).

Antioxidants are considered one of the most important traits deciding the quality of the wheat grains. They have many health benefits, such as boosting the immune system, protecting from various pathogenic and auto-regulatory diseases (Ranjbar et al., 2019). They scavenge the free radicals present inside the cells and balance the redox system of the cells. The accumulation of antioxidants such as tannin, vitamin E, and total phenolic compounds is considered a desirable trait for good quality grain and flour (Goswami et al., 2020). Wheat *cvs*. have shown wide diversity in their antioxidant content (Yadav et al., 2021). Wheat grains have very significant amounts of phenolic and flavonoids with very high bioavailability, as compared to other nutrients (Ma et al., 2021). An increase in the accumulation of antioxidants above the optimum level causes bitterness and leads to compromised quality and taste (Bruno et al., 2019). Antioxidants in the wheat grains are good from a health point of view. Other than the health benefits, antioxidants have been reported to be used as food preservatives and play a very important role in enhancing the quality and shelf-life of the flour (Chen et al., 2021). Anti-nutritional factors, such as phytate in flour, have been reported to reduce the bioavailability of micronutrients such as iron and zinc, and are considered a nutrient that reduces the quality of flour (Alkarawi et al., 2018). The activity of phytases has been reported to be reduced in wheat and is one of the reasons behind the significant accumulation of phytate in grains (Madsen and Brinch-Pedersen, 2020). Phytase activity was observed to be more stable in a ground sample of wheat than in whole grains (Schollenberger et al., 2022). The regulation underlying the phytase activity and accumulation of phytate is not well understood.

Protein thiols are present in the cytosol in their reduced state. In most cells, the cytoplasm is a reducing environment, and protein thiols are maintained in their reduced state. Thiol redox buffers maintain the reduced state of thiol in cells (Meyer and Hell, 2005). Reactive oxygen species (ROS) are highly reactive molecules containing oxygen, such as superoxide, hydrogen peroxide, and hydroxyl radicals. They are produced as byproducts of normal cellular metabolism, and also arise from different biotic and abiotic stresses. ROS are involved in damaging cellular components such as lipids, proteins, and DNA, leading to oxidative stress, which has an adverse effect on the growth and development of plants. ROS and other free radicals damage the cellular compartment, organelles, enzymes, nucleic acids, and proteins inside cells. In order to neutralize these free radicals, different defense mechanisms become active inside the cells and are triggered by redox-sensitive transcription factors and cysteine residues present in the respective proteins. Cysteine (Cys) thiol groups have been reported to have a acid dissociation constant (pKa) of 8, which causes more reactive thiolate (RS−) and anion formation at physiological pH. The modification of thiol causes up- or downregulation of TFs, which ultimately regulates the expression of different genes and proteins involved in various metabolic pathways (Li et al., 2023). Thiol has been reported to indirectly regulate the expression at the transcript and protein level, as evident from the changes in the cellular response and metabolites.

Active thiols are oxidized by ROS, especially H_2_O_2_, which is produced in response to different environmental and cellular conditions. Thiol has intrinsic pKa values of 8.4–8.6. The catalytic Cys groups in thiol have lower pKa values of 5.5–7.4, which in turn act as a H_2_O_2_-sensing center and react with H_2_O_2_ under physiological conditions (Chae et al., 2023). The thiol group undergoes oxidation at the H_2_O_2_ level and forms an inter or intramolecular disulfide bond (–S–S–), sulfenic acid (–SOH), sulfinic acid (–SO_2_H), and sulfonic acid (–SO_3_H), which alters the structure, activity, and interactions of different proteins and enzymes and ultimately changes their localization and regulates the metabolism and physiology of plants. Similarly, thiol peroxidases such as thioredoxin peroxidases (TPXs) and glutathione peroxidases (GPXs) are also oxidized by H_2_O_2_, especially the cys residue, and are oxidized into different forms and transfer their redox signal to target genes and proteins (Paulsen and Carroll, 2013). Thioredoxin (Trx) and glutaredoxin (Grx) proteins depend upon thiol peroxidase for their oxidizing potential and receive electrons from NADPH/GSH with the help of thiol reductases (Meyer and Hell, 2005). Trx/Grx proteins can act as redox sensors in both oxidized and reduced forms in response to different cellular and environmental conditions (Paulsen and Carroll, 2013).

Various enzymes have been reported to interplay in the spatiotemporal mode in carbon assimilatory processes, especially photosynthesis and starch biosynthesis. The developing endosperm has also been reported to have a diverse network of enzymes working in tandem to synthesize starch to be packed inside the endospermic tissues. The accumulation of macro- and micronutrients is influenced by the activities of pathway-associated enzymes, other than environmental factors, and is one of the decisive factors in the nutritional composition of grains (Zhang et al., 2021). Storage protein in seeds basically acts as a reservoir of nitrogen, carbon, and sulfur. The main objective of the plant when storing the SSPs is to use them during germination or in adverse conditions. Wide diversity has been reported in the nature and composition of SSPs in the grains of different plant species (Antonets et al., 2020).

Here, we have studied the effect of the regulation of thiol on the accumulation of macro- and micronutrients inside the grains of diverse genotypes of wheat. We have established a correlation between thiol and the proximate composition in the grains. The findings can be used to evaluate diverse genotypes of wheat for grain quality and for the development of nutrient-dense grains.

## Materials and methods

### Samples and growing conditions

We have used 16 diverse genotypes of wheat selected from the core set developed for improved grain quality at the ICAR-Indian Agricultural Research Institute (ICARI), New Delhi. The list of wheat genotypes used in the present investigation has been presented in the Supplementary Material (**Supplementary Table S1**). The pre-treated seeds were sown in pots (22.5 cm diameter) at the Nanaji Deshmukh Plant Phenomics Centre, Pusa, ICAR-IARI, New Delhi, and recommended intercultural operations were followed along with the timely application of fertilizers and irrigations. The pots were kept in a regulated glasshouse at a temperature of 22 ± 3°C (daytime) and 18 ± 3°C (nighttime) during the vegetative stage, and at 28 ± 3°C (daytime) and 32 ± 3°C (nighttime) during the pollination and grain-filling stages. Other parameters, such as light and RH, were maintained, as mentioned in our previous publication (Kumar et al., 2022). Only healthy plants were tagged and further used for sample collection at a specific stage based on the Feekes scale. Samples were collected in three biological replicates from the tagged plants and were packed in an airtight container and stored at -20°C for further downstream analysis.

### Determination of free thiol content

The freshly harvested grains from different genotypes of wheat were further subjected to fine grinding using a mechanized mini-grinder machine (Kolar Mill Stores Pvt. Ltd. Karnataka, India), keeping the temperature constant in order to protect them from the adverse effects of milling temperature on biochemical constituents. We used Ellman’s test to estimate the free thiols in the ground sample (Ellman, 1959). In brief, 10 µL of powder extract (prepared by passing the slurry prepared in green solvent through a chromatographic column) was placed in a test tube along with 50 µL of Ellman’s reagent [50 mM of sodium acetate (NaoAc), 2 mM of 5’5-Dithio-bis-(2-nitobenzoic acid) in water, and 1M of Tris solution (pH 8.0)], 100 µL of Tris solution, and 840 µL of distilled water. The absorbance of the solution was taken at 412 nm using a UV-Vis spectrophotometer. The thiol content was calculated using the following equations:


$$Abs_{sample}=\left(Total\;volume/Sample\;volume\right)\times \left(Abs_{412}\right)$$

$$Free\;Thiol\;\left(mM\right)=Abs_{sample}/13600$$

The thiol content was expressed as µmol/g of the sample.

### Expression analysis of thiol-reducing genes

The total RNA was extracted from the developing seeds (high thiol content - Halna, HD2985; Low thiol content - NIAW34, PBW443) collected during the button (G_0_), milky-ripe (G_1_), and seed hardening (G_2_) stages using a RaFlex Total RNA Isolation Kit (GeNei, USA). The quality of the isolated total RNA was checked using a BioAnalyzer (Agilent, USA), and an OD 260 to 280 ratio of > than 1.9 was used for the expression analysis. We have selected the thiol-reducing genes [Thioredoxin (*TRX*, acc. no. AF438359.1), Glutaredoxin (*GRX*, acc. no. AF542185.1), and Glutathione reductase (*GR*, acc. no. XM_044564013)] for the oligo design using the Genefisher2 Primer design software (https://bibiserv.cebitec.uni-bielefeld.de/genefisher2/) and it was synthesized commercially (**Table 1**). cDNA was synthesized using the Revert^Aid^ H Minus First Strand cDNA Synthesis Kit (Thermo Fisher Scientific) following the protocol, as given by the manufacturer. The expression analysis was performed using the Bio-Rad CFX96 platform, and the steps were followed as mentioned in our previous publication (Kumar et al., 2022). We used the β-actin gene (acc. no. JQ004803.1) as the endogenous control gene for normalizing the C_t_ value. The relative fold expression (2^^-ddCt^ value) was calculated using the Pfaffl method (Pfaffl et al., 2002).

**Table 1:** List of primers used for the expression analysis of genes linked with thiol-based redox sensing in wheat.

Oligo’s ID	Primer sequence (5’-3’)	Tm (°C)
TRX-F	5’-GGCAAGGATCGTAGCATTGT-3’	58.3
TRX-R	5’-CGGCATAAACAGGTGCAATG-3’	58.0
GRX-F	5’-ATTGCACTGAACAAGGGAGG-3’	58.0
GRX-R	5’-GCCAAACACATCCAACTTCG-3’	57.9
GR-F	5’-GAAGATGCTCAAGGACAGGG-3’	57.9
GR-R	5’-CAATCCGAGCTGATAGGGTG-3’	57.8
Act-F	5’-CCTGGTATACACGAAGCGAC-3’	58.1
Act-R	5’-GGAAAGTGCTAAGAGAGGCC-3’	57.9

*TRX, thioredoxin gene; GRX, glutaredoxin gene; GR, glutathione reductase gene; Act, actin gene.

### Cysteine and methionine estimation in regulatory genes associated with different metabolic pathways

In order to know the abundance of cysteine and methionine (Met) amino acids residues in regulatory genes linked with different metabolic pathways associated with the biosynthesis of yellow pigment and other proximate composition (macro- and micronutrients), we retrieved the amino acid sequences of the following genes from NCBI that are involved in different biosynthesis pathways and have been cloned from contrasting wheat *cvs*.: phytoene synthase gene (*PSY*, NCBI acc. no. FJ393546), which is involved in yellow pigment synthesis; ADP glucopyrophosphorylase gene (*AGPase-L*, acc. no. DQ406820), *Triticum aestivum* ADP glucopyrophosphorylase-1 gene (*TaAGPase-1*, acc. no. KC347594), starch synthase (*SS*, acc. no. AY050174), Starch synthase-III (*SS-III*, acc. no. AF258608), and pullulanase gene (acc. no. XM_044583776), which are involved in the starch biosynthesis pathway; phytoene desaturase (*PDS*, acc. no. FJ517553) and β-Carotene hydroxylase (*BCH*, acc. no. JX171675), which are involved in the tannin biosynthesis pathway; phenylalanine ammonia lyase gene (*PAL*, acc. no. XM_044557325), which is involved in the biosynthesis of phenolic compounds; chalcone synthase 2-like (*CHS*, acc. no. XM_044543986) and inositol hexakisphosphate kinase gene (*IP6K1*, acc. no. XM_044517289), which are involved in the biosynthesis of phytic acid. Further, the amino acid sequences were characterized using the Prot pi tool available online (https://www.protpi.ch/Calculator/ProteinTool). The abundance of Cys/Met was presented as a percentage of the amino acids.

### Cys/Met estimation in the grains of contrasting wheat *cvs.* using high-performance liquid chromatography

We estimated the Cys/Met in the grains following the standardized method at our Nutrition Analysis Lab, ICAR-IARI, New Delhi. In brief, 50 µg of the sample was taken and transferred into a clean glass vial. To 50 mL broth tubes, 20 mL of 6N HCl was added, and the glass vial containing the sample was dipped into the tube and sealed with parafilm. The tube was placed in a dry bath at 60°C under N_2_ gas for 15 min to maintain inertness. Further, the temperature was increased to 110°C and it was incubated for 24 h for complete hydrolysis of the protein sample (Marino et al., 2010). The filtrate was then evaporated in a vacuum flash evaporator and was made acid free by repeated washing with distilled water and subsequent evaporation. The hydrolyzed sample was further subjected to pre-column derivatization using O-phthalaldehyde in the presence of βxA7B5;-mercaptoethanol. Further, 80 µL of the derivatized sample was injected in high-performance liquid chromatograph (HPLC) (Shimadzu, Model CBM 20 A) equipped with a C18 reverse phase (RP) column and a fluorescence detector. The amino acids were identified and quantified by comparing the retention times and peak areas with those of the Sigma standard. The concentration of amino acids was presented as the percentage of a mole.

### Yellow pigment estimation

The estimation of yellow pigment (β-carotene) in the sample was carried out as per the modified method of Kumar et al (2022), published in the technical bulletin of ICAR-Indian Agricultural Research Institute, New Delhi, India. We used 1 kg of treated wheat grains (conditioned to 13% moisture and debranned to 6% using a fabricated peeling machine available at the Agricultural Engineering Workshop at ICAR-IARI, New Delhi, India). We used 40 g of fine powdered (100 mesh size particles) from each genotype prepared using a mini-miller machine (Kolar Mill Stores Pvt. Ltd. Karnataka, India). The fine powder was stored in air-tight bags in two groups for further downstream characterization. The estimation of total yellow pigment was carried out by using the cold percolation method. The fine powdered flour (40 g) was loaded onto the chromatograph column packed with cotton and sodium sulphate. Further, 75 mL of n-butanol was used for the extraction of the eluent from the bottom in a fresh test tube. The extract was packed in dark colored bottle and was used for the estimation of yellow pigment by measuring the absorbance at 412 nm. The yellow pigment was calculated using the extinction coefficient of 13,000 mM cm^-1^.

### Estimation of total starch and resistant starch

The collected samples (0.1 g) were fractionated in 80% ethanol (hot) as per the protocol of Thayumanavan and Sadasivam (1984). In brief, the residue retained after centrifugation was washed repeatedly until the absence of color formation by the anthrone reagent. The dried residue was mixed with 5.0 mL of water and 6.5 mL of perchloric acid (52%). The mixture was incubated at 0°C for 20 min followed by centrifugation at 12,000 rpm for 10 min. The extraction using fresh perchloric acid was repeated twice, and further volume was made up to 100 mL. An aliquot of 0.1 mL was taken, and the volume was made up to 1 mL with water. Further, 4 mL of anthrone reagent was added, and the reaction mixture was heated for 8 min in a boiling water bath, followed by rapid cooling. The intensity of the green to dark green color was read by measuring OD at 630 nm. The glucose standard was used to estimate the concentration of starch by multiplying it by a factor of 0.9.

RS in the grain was estimated using the Resistant Starch Assay kit (Megazyme) (Englyst et al., 1992). In brief, 50 g of grains were used for the estimation following the protocols as provided by the manufacturers. A mixture of 0.1 mL of aliquot and 3.0 mL of glucose oxidase/peroxidase (GOPOD) reagent was incubated at 50°C for 20 min, and the OD was read at 510 nm against the reagent blank. The RS was calculated using the D-glucose solution standard provided in the kit.

### Estimation of total phenolic content

The total phenolic content was estimated using the method of Singleton et al. (1999). In brief, 0.5 mL of supernatant (extract from the slurry by passing the mixture through a chromatograph column) was mixed with 5 mL of 1 M Folin–Ciocalteu reagent (FCR). Further, the solution was neutralized by adding 4 mL of 75 g/L saturated sodium carbonate and the solution was kept at room temperature (RT) for at least 2 h. The solution was further used for measuring the absorbance at 765 nm. The total phenolic content (TPC) was measured using Gallic acid as a standard curve, and the result was presented in terms of Gallic acid equivalents (mg GAE/g DW).

### Estimation of total tannin content

The tannin content was estimated using the Folin–Ciocalteu reagent method (Everette et al., 2010). We used 0.1 mL of extract and a further reaction mixture was prepared by adding 0.5 mL of FCR, 1 mL of 35% sodium carbonate (Na_2_CO_3_), and 7.5 mL of double-distilled water, followed by incubation at RT for 30 min. The RM was further used to measure the absorbance at 700 nm against the blank. The total tannin content was calculated using the standard curve of tannic acid and expressed as mg/mL.

### Estimation of micronutrients (iron and zinc)

The finely ground grains (1.0 g) were digested using 20 mL of the digestion mixture (consisting of HNO_3_ and HClO_4_ in the ratio of 9:4) using the digestion chamber. The mixture was evaporated until the volume was reduced to 3 to 5 mL. The flasks were then cooled at room temperature, and double-distilled water was added to make the volume 50 mL. The clear, digested solution in each flask was filtered using Whatman filter paper No. 42 and used to analyze the micronutrients. The micronutrients Zn and Fe were analyzed using an atomic absorption spectrophotometer (AAS, Electronics Corporation of India Limited, Hyderabad, India) by measuring the absorption at 213.9 nm and 248.3 nm for Zn and Fe, respectively. The concentration was estimated using the pure standard and the content was expressed as μg/g DW.

### Estimation of anti-nutritional factor (phytic acid) and phosphorus

The finely ground grains (1 g) were used for the phytic acid estimation by digesting the powder using 20 mL of hydrochloric acid (0.66 M) overnight. Further, 0.5 mL of the extract was neutralized by adding 0.5 mL of NaOH (0.75 M) and used for the enzymatic dephosphorylation reaction using the phytic acid assay kit (Megazyme). The supernatant was used for the colorimetric determination of phosphorus by measuring the absorbance at 655 nm and the calculation was conducted as given in the instruction manuals.

### Estimation of total storage protein and gluten

The crude nitrogen present in the grain was estimated using the micro-Kjeldahl method (Miller and Houghton, 1945). In brief, the flour (100 mg) was digested in strong sulfuric acid (10 mL) along with 2 g of K_2_SO_4_ and CuSO_4_ (in a ratio of 10:1) for 90 min at 420°C, followed by distillation using 40% NaOH and 4% Boric acid. The trapped ammonia was determined through titration with 0.1 N HCl and the burette reading was used to calculate the percentage of nitrogen using the formula -


$$\begin{array}{c} \%\;N=14.01\times Burette\;reading\times 100\times Normality\;\left(HCl\right)/1007\\ \times Volume\;of\;sample\;taken \end{array}$$

The percentage of nitrogen calculated was used for the calculation of total protein content using the conversion factor of 5.81.

The gluten was estimated in the finely ground flour following the traditional method of washing the dough with water. We used 2 g of flour and added 1–2 mL of water for dough preparation. The prepared dough was wrapped in two layers of chicken cloth and washed with distilled water until a fine elastic layer was left over. The gluten leftover was dried in an oven at 72°C for overnight, and further weighed for the calculation of the percentage of gluten in the flour.

### Statistical analysis

The samples were three biological replicates for the biochemical analysis, and we used the statistical tool of one-way ANOVA to analyze the variance of different parameters for statistically significant differences (P ≤ 0.05). The correlation between the biochemical traits was analysed using Pearson’s correlation coefficient (r, p<0.01).

## Results and discussion

### Total thiol and disulfide profiling in the grains of diverse genotypes of wheat

**Figure 1:**
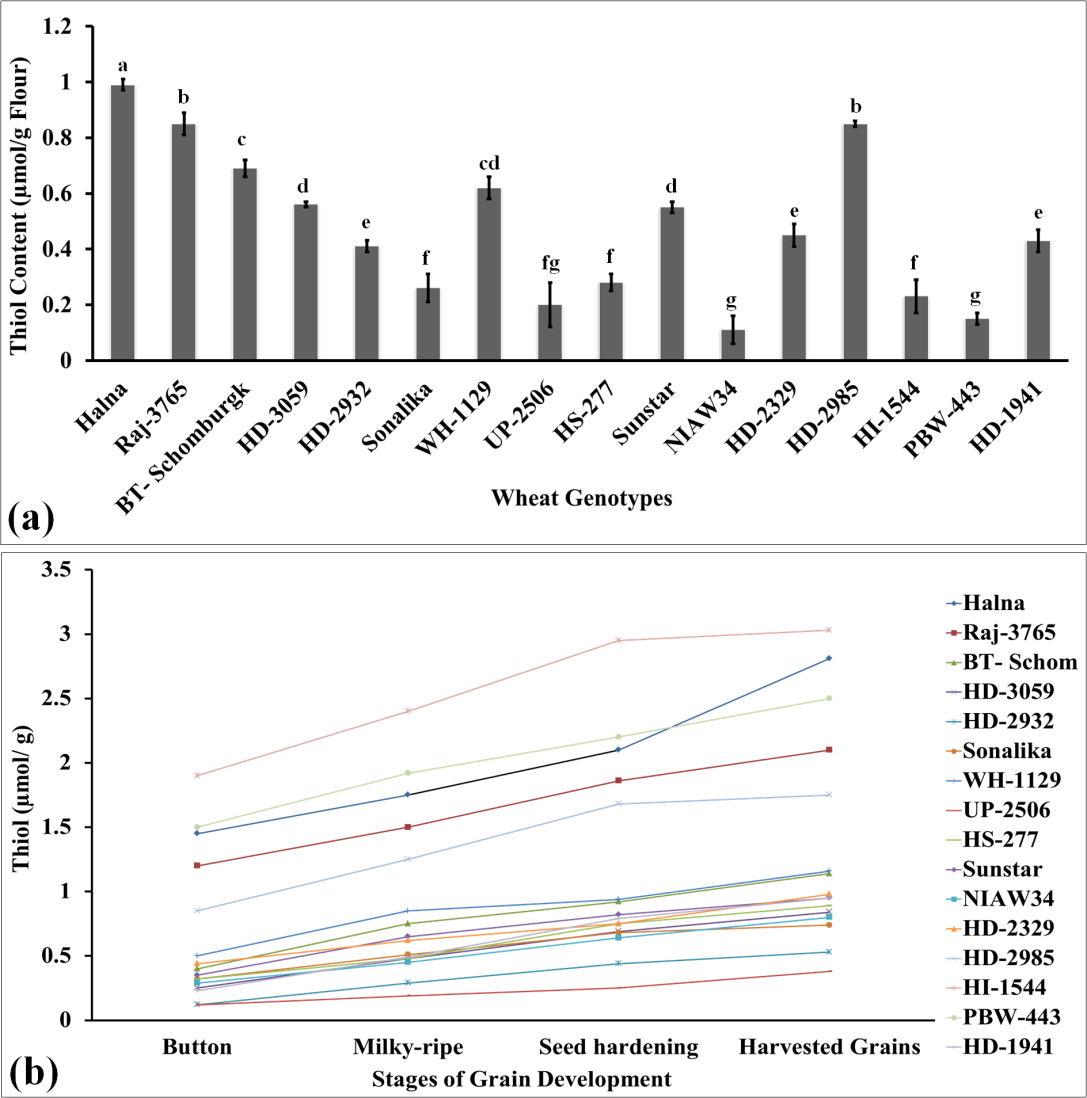
Thiol profiling in the developing endospermic tissues and harvested grains of diverse genotypes of wheat grown under regulated conditions. **(a)** Total thiol content in harvested grains. **(b)** Variations in the thiol content in developing endospermic tissue. Sub-stages of endosperm used for the estimation include the button, milky-ripe, seed hardening, and harvested grain stages. In total, 16 diverse genotypes of wheat were selected for the study. The vertical line above the bar denotes the *SEM* (n=3); a letter above a bar shows the significance level (P<0.05).

Thiol is a very important component of storage proteins, providing stability and strong networking in dough and many desirable rheological characteristics in flour. It is considered one of the desirable traits for analyzing the quality of flour. We have analyzed the thiol content in the grains of diverse genotypes of wheat and observed the highest thiol content in the grain of wheat *cvs*. Halna followed by HD2985 and Raj3765 (**Figure 1a**). The thiol content was observed to be th elowest in wheat cv. NIAW34.

In order to understand the effect of the changes in the thiol content on developing endosperm tissues, we collected samples at different grain-filling stages: button, milky-ripe, seed hardening, and mature grains, and used them for the thiol estimation (**Figure 1b**). Wheat cv. Halna had the highest thiol content at different sub-stages of endosperm development, as compared to other *cvs*., whereas the lowest was observed in PBW343 and NIAW34 during different sub-stages. We observed significant variations in the flour thiol content across the genotypes (*F*-values are significant at p ≤ 0.05). Most of the wheat *cvs*., which are popular among consumers for flour and chapatti-making quality (HD2985, Raj3765, etc.), had very high thiol content, as compared to other genotypes. Manu and Rao (2008) reported that high thiol content in flour decreases the toughness of chapatti.

**Figure 2:**
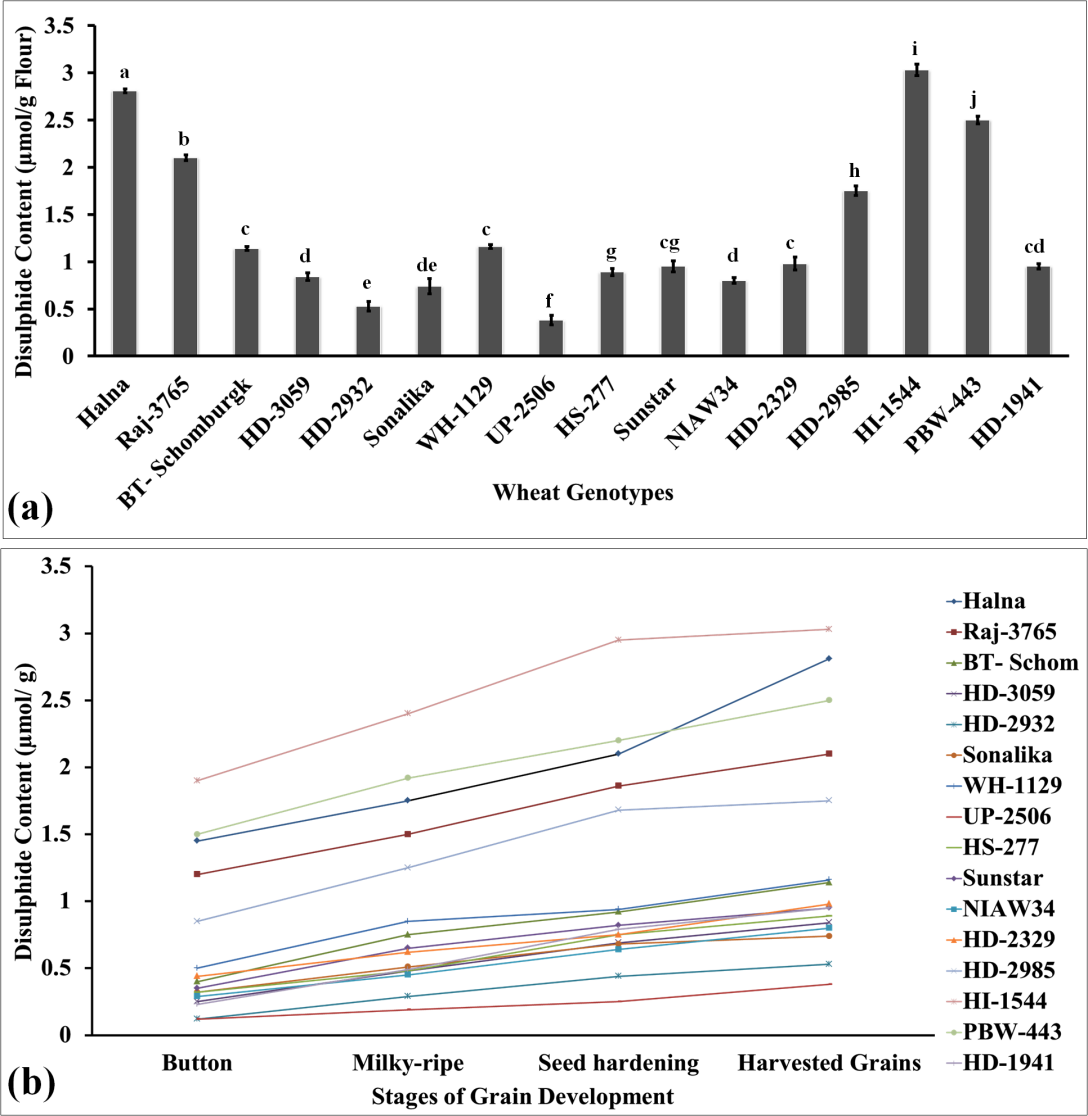
Disulfide profiling in the developing endospermic tissues and harvested grains of diverse genotypes of wheat grown under regulated conditions. **(a)** Total disulfide content in the harvested grains. **(b)** Variations in the disulfide content in the developing endospermic tissue. Sub-stages of endosperm used for the estimation include the button, milky-ripe, seed hardening, and harvested grain stages. In total, 16 diverse genotypes of wheat were selected for the study. The vertical line above the bar denotes the *SEM* (n=3); a letter above a bar shows the significance level (P<0.05).

Disulfide bonds (SS) are formed by the oxidation of sulfhydryl groups (SH) and have been reported to be involved in stabilizing the folded conformation of gliadin (monomeric proteins). The polymeric structure of dough is also stabilized due to the presence of disulfide bonds. We analyzed the disulfide bonds in the grains of diverse genotypes of wheat. The disulfide content was observed to be the highest in the grains of wheat cv. HI1544 followed by Halna and PBW443 (**Figure 2c**). The lowest disulfide content was observed in wheat cv. UP-2506. Disulfide bonds are linked with the empirical rheology of flour and are considered one of the important traits.

We analyzed the pattern of increase in the disulfide content in the developing endosperm tissue in diverse *cvs*. and observed the highest disulfide content in wheat cv. HI1544 and the lowest in UP2506 at different sub-stages of endosperm growth and development (**Figure 2b**). Manu and Rao (2008) observed that protein disulfide content showed a positive correlation with dough hardness and the texture of chapatti. The thiol content has been observed to influence the formation of disulfide bonds in flour and helps in improving the rheological characteristics of dough. Various studies have shown that GSH-dependent protein-disulfide oxidoreductase (TPDO) present in the developing wheat kernels manipulate the quality matrix of dough, causing stiffness. Disulfide bond dynamics have been reported to enhance the dough extensibility in wheat (Osipova et al., 2007).

### Expression analysis of genes responsible for thiol-based redox sensing

**Figure 3:**
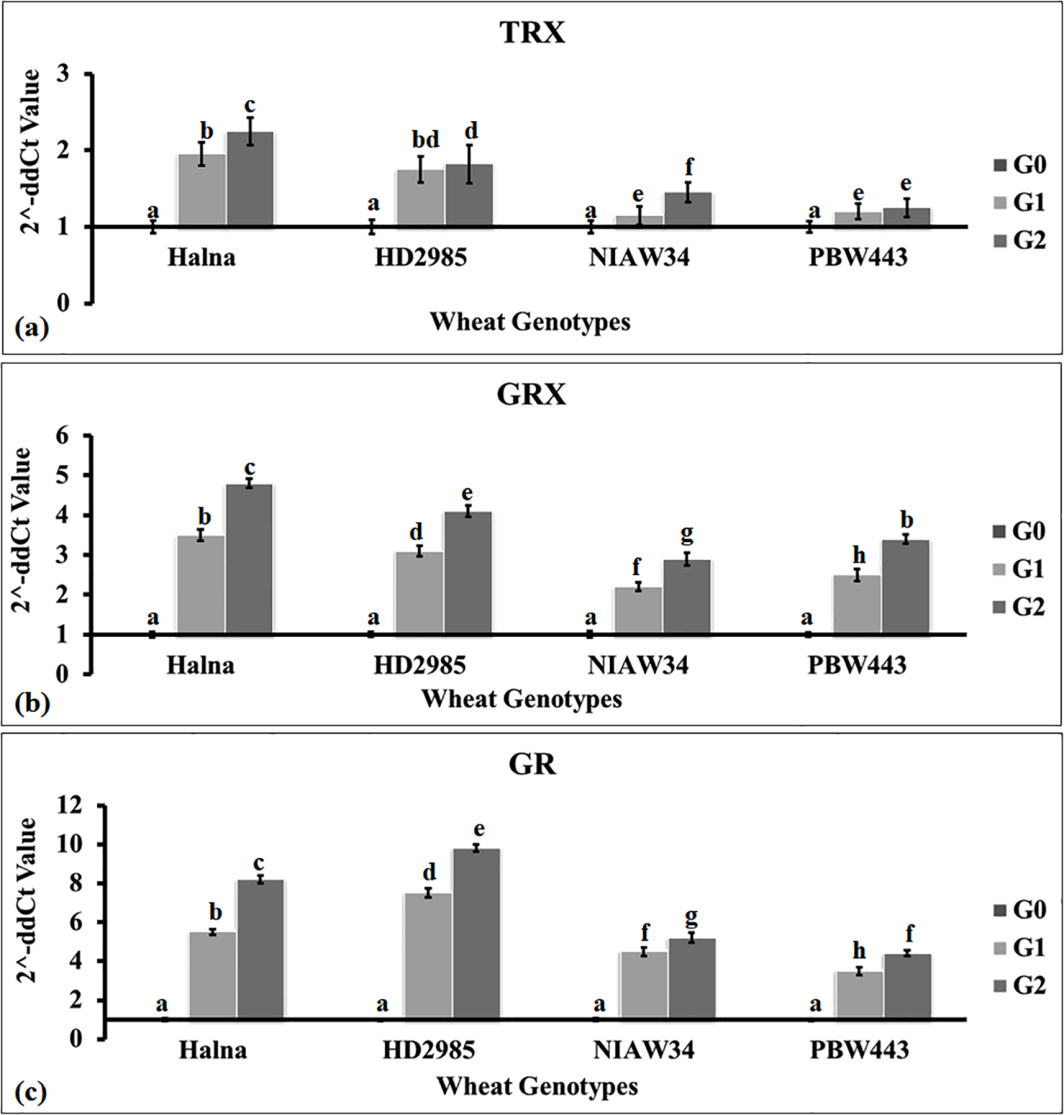
Expression profiling of genes linked with thiol-based redox sensors in developing endospermic tissue of wheat. **(a)** Expression of the thioredoxin (*TRX*) gene. **(b)** Expression of the glutaredoxin gene (*GRX*). **(c)** Expression of the glutathione reductase gene (*GR*). Four genotypes were selected for the expression profiling: high thiol content in grains: Halna and HD2985; low thiol content in grains: NIAW34 and PBW443. The sub-stages of endosperm tissue used for the expression analysis were the button (G_0_), milky-ripe (G_1_), and seed hardening (G_2_) stages. The vertical line above the bar denotes *SEM* (n=3).

We selected three genes—*TRX* (acc. no. AF438359.1), *GRX* (acc. no. AF542185.1), and *GR* (acc. no. XM_044564013)—to assess the thiol-based redox sensing in developing endosperm (sub-stages G_0_, G_1_, and G_2_) of Halna and HD2985 (high-thiol content genotypes) and NIAW34 and PBW443 (low-thiol content genotypes). *TRX* expression was observed to be higher (2.4-fold) in wheat cv. Halna during the G_2_ stage compared to the G_0_ stage (**Figure 3a**). The expression was observed to be the lowest in wheat cv. PBW443 compared to other genotypes. Overall, we observed an abundance of *TRX* transcripts in the developing grains of genotypes with high thiol content (Halna and HD2985) compared to low thiol-containing genotypes (NIAW34 and PBW443).

We observed similar patterns of GRX expression in contrasting wheat genotypes (**Figure 3b**). The relative fold expression was observed to be the highest (4.9-fold) in wheat cv. Halna during the G_2_ stage, whereas it was observed to be the lowest (3.1-fold) in cv. NIAW34 compared to G_o_. Sainz et al. (2022) reported the differential expression of 12 *Trx* and 12 *Grx* genes in response to water deficit stress in soybean.

Similarly, expression analysis of GR showed a relatively higher expression (9.9-fold) in wheat cv. HD2985 during the G_2_ stage compared to G_0_ (**Figure 3c**). The GR expression was observed to be the lowest (4.1-fold) in wheat cv. PBW443 during the G_2_ stage compared to G_o_. We observed significant variations (p ≤ 0.05) in the expression of *TRX*, *GRX*, and *GR* during different sub-stages of endosperm development in contrasting wheat *cvs*., with an abundance of transcripts in Halna and HD2985 compared to NIAW34 and PBW443. He et al. (2023) identified 85 GRX genes in common wheat using a bioinformatic method and reported differential upregulation of these genes under biotic and abiotic stresses in wheat.

### Cys/Met estimation in regulatory genes associated with different metabolic pathways

**Figure 4:**
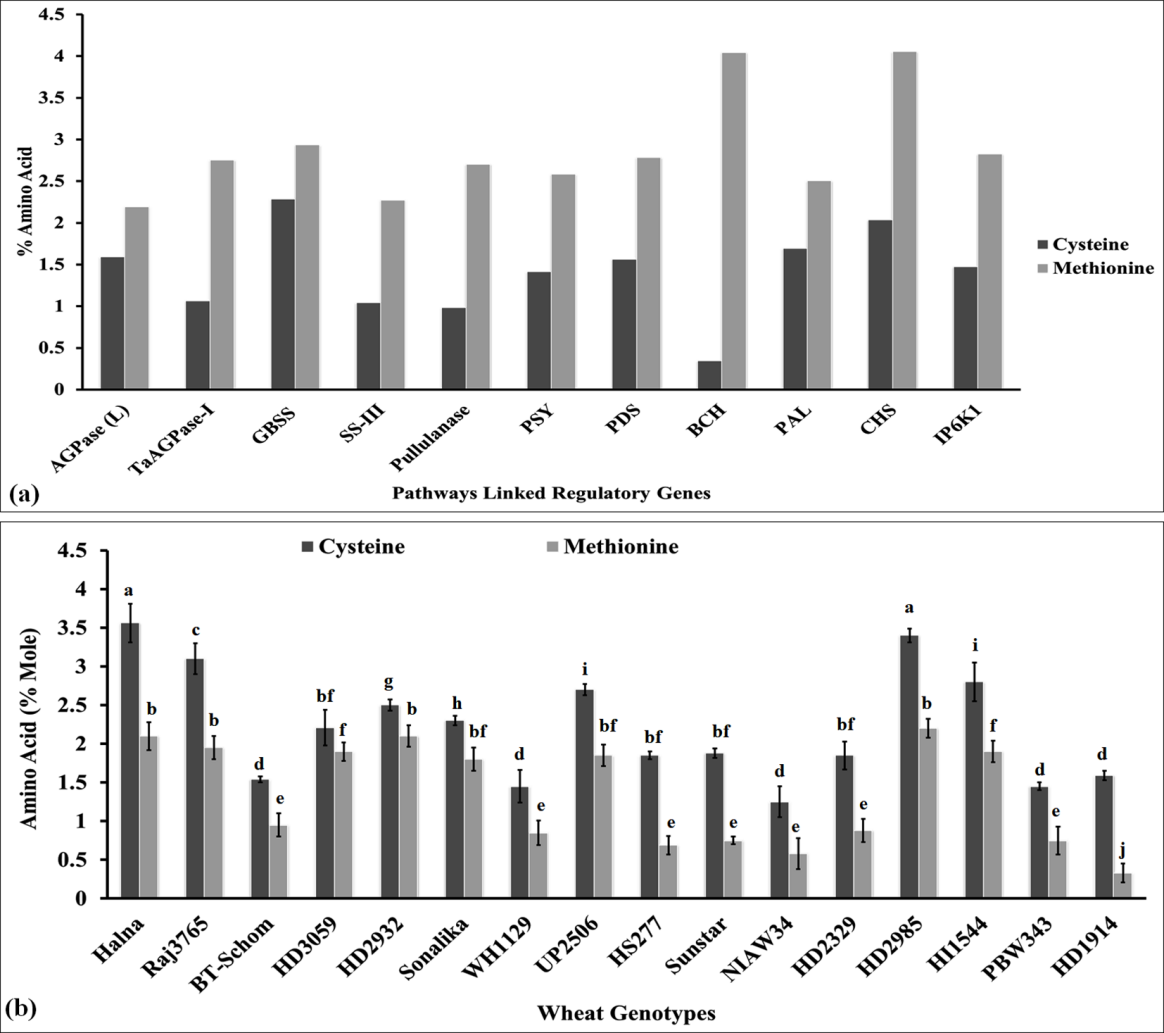
Characterization of sulfur-containing amino acids in the key regulatory genes linked with various metabolic pathways and in the grains of diverse genotypes of wheat. **(A)** Cys/Met estimation in regulatory genes associated with different metabolic pathways. **(B)** Cys/Met estimation in the grains of diverse genotypes of wheat. In total, 11 genes linked with key metabolic pathways were retrieved from NCBI, and their amino acid compositions were analyzed using the Prot pi tool. Moreover, 16 diverse genotypes of wheat were selected for the Cys/Met estimation in grains. The vertical line above the bar denotes *SEM* (n=3); a letter above a bar shows the significance level (P<0.05).

The ratio of Cys: Met has been reported to play very important role in deciding the structural and thermal stability of protein under oxidizing conditions (Bhopatkar et al., 2020). In order to analyze the Cys/Met ratio, we retrieved the amino acid sequences of some of the regulatory genes (cloned from *Triticum aestivum*) linked with different metabolic pathways associated with the biosynthesis of macro- and micronutrients in the grains, i.e., yellow pigment biosynthesis (*PSY*, acc. no. FJ393546), starch biosynthesis [*AGPase-L*, (acc. no. DQ406820), *TaAGPase-1* (acc. no. KC347594), *SS* (acc. no. AY050174), *SS-III* (acc. no. AF258608), and pullulanase gene (acc. no. XM_044583776)], tannin biosynthesis [*PDS* (acc. no. FJ517553) and *BCH* (acc. no. JX171675)], phenolic compound biosynthesis (*PAL*, acc. no. XM_044557325), and phytic acid biosynthesis [*CHS* (acc. no. XM_044543986) and *IP6K1*, (acc. no. XM_044517289)]. The amino acid sequences were characterized for the estimation of Cys/Met in the amino acid pool (**Figure 4A**). GBSS is considered one of the important enzymes involved in the starch biosynthesis pathway. We observed 2.25% Cys and 2.95% Met in the amino acid sequence of *GBSS* cloned from wheat (**Figure 4A**). Similarly, in the case of pullulanase, which is involved in RS synthesis, it had 0.98% Cys and 2.7% Met in the amino acid pool. The Cys content was observed to be the highest (2.25%) in *GBSS* and Met was observed to be the highest (4.04%) in the *BCH* gene involved in tannin synthesis. The ratio of Cys: Met was observed to be the highest in *GBSS* (0.77) and the lowest in *BCH* (0.08). Other genes also showed the same pattern of Cys and Met in their amino acid sequences. All these key genes were observed to have a high percentage of Cys and Met, which provide stability to the proteins against various factors. Walker et al. (2022) reported that S-containing amino acids protect the proteins from oxidation and correlated it with the folding stabilities of the proteins under stress.

### Cys/Met estimation in the grains of contrasting wheat *cvs* using HPLC

We estimated the Cys and Met concentration in the grains of diverse *cvs*. of wheat in order to understand its effect on different metabolic pathways linked with various macro- and micronutrients. The cysteine content was observed to be the highest (3.56% mole) in wheat cv. Halna (thermotolerant) followed by HD2985 (3.40% mole), whereas it was observed to be the lowest (1.25% mole) in NIAW34 (thermosusceptible) (**Figure 4B**). Similarly, the methionine content was observed to be the highest (2.20% mole) in wheat cv. HD2985 (thermotolerant) and to be the lowest (0.33% mole) in cv. HD1914 (thermosusceptible). We observed significant (p ≤ 0.05) variations in the Cys and Met content in the grains among the diverse genotypes. Most of the genotypes that have been reported to be thermotolerant, such as HD2985, Halna, and HD2932, showed very high Cys and Met contents, whereas thermosensitive *cvs*., such as BT-Schomburgk, HD1914, and PBW343, showed low Cys and Met contents in the amino acid pool of the grains. The abundance of Cys and Met may be the reason behind the high stability of the proteins and tolerance level of the plant under heat stress, since it behaves as a strong antioxidant, enhancing the stability of the enzymes involved in various pathways responsible for the synthesis of many macro- and micromolecules. Liu et al. (2020) reported that higher concentrations of glutathione maintain the redox balance and enhance the accumulation of protein and S-containing amino acids in the grains of maize, which in turn improves the grain quality.

**Figure 5:**
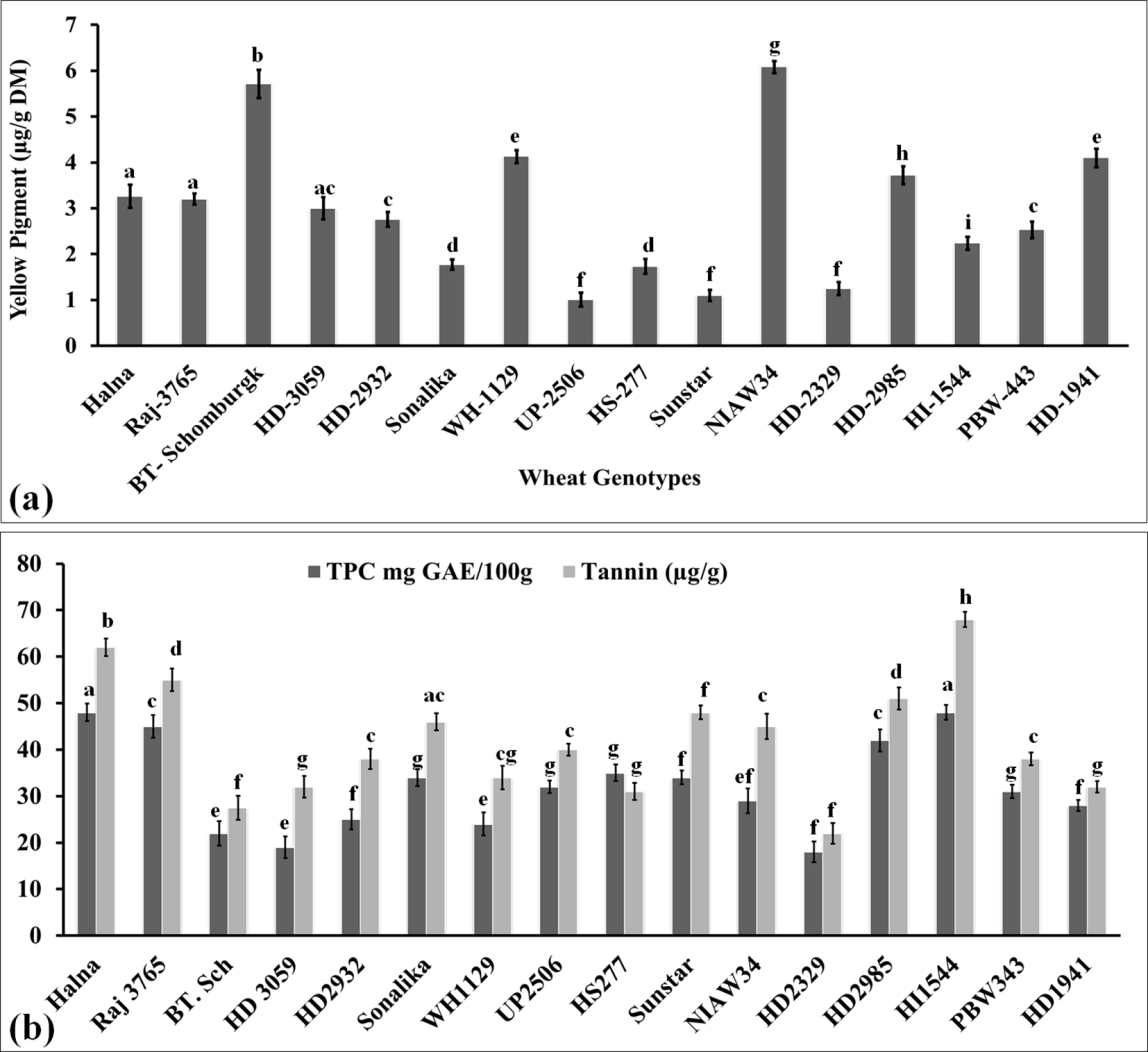
Yellow pigment and antioxidant estimation in the grains of diverse genotypes of wheat. **(a)** Yellow pigment content in the grains. **(b)** Total phenolic and tannin content in the grains. In total, 16 diverse genotypes of wheat were selected for the study. The vertical line above the bar denotes the *SEM* (n=3); a letter above a bar shows the significance level (P<0.05).

Yellow pigment is essentially carotenoids such as lutein and α/βxA7B5;-carotene, and these are considered potential natural antioxidants with many medical benefits. It also provides the shining color of the grains and is one of the traits used to decide the export value of wheat grains. This is one of the deciding factors in the popularization of wheat grains and consumer preference. Many processed products require wheat grains to have a very high content of yellow pigment. We also analyzed the yellow pigment in the grains of diverse genotypes of wheat to decide their quality. The yellow pigment content was observed to be the highest in the grains of wheat cv. NIAW34 (6.08 µg/g dry matter), followed by BT-Schomburgk (5.71 µg/g dry matter), whereas the content was observed to be the lowest in wheat cv. UP-2506 (1.01 µg/g dry matter) (**Figure 5a**). We observed significant variations (p ≤ 0.05) in the total yellow pigment content in the diverse genotypes selected for the present study. Butler and Ghugre (2020) reported that β-carotene acts as a potential iron enhancer and limits the phytate-chelating mechanism. It causes an increase in the availability of ionizable iron, which is considered an indicator of the bioavailability index. Wheat genotypes with a high yellow pigment are recommended to be used in the pasta industry, and it is one of the desirable traits.

### Variations in the grains’ antioxidants

#### Accumulation of total phenolic content

Phenolic is considered one of the important antioxidants in the grains, which has many beneficial effects, such as helping in scavenging the free oxygen radicals inside the cells and protecting the proteins, enzymes, and organelles from disintegration. We analyzed the phenolic content in diverse wheat *cvs*. and observed the highest TPC in Halna (48.5 mg GAE/100g) and HI1544 (49.2 mg GAE/100g), whereas the lowest content was observed in HD2329 (20.2 mg GAE/100g) (**Figure 5b**). Most of the popular wheat *cvs*., such as Halna, HD2985, Raj3765, Sonalika, showed higher accumulation of TPC compared to other *cvs*. Ali et al. (2023) highlighted the importance of phenolics in improving the grain quality of pearl millet. Phenolics have also been reported to suppress the activity of many starch-degrading enzymes inside the human body and, in turn, provide a low spike of glucose and make it suitable for consumption by diabetic patients (Deka et al., 2022).

#### Accumulation of tannin content

Tannin is a water-soluble polyphenol and is considered one of the most effective antioxidants within the plant system. It provides the bitterness and astringency in the seeds and helps in protecting the grains from pests. It reduces cholesterol and lowers blood pressure inside the human body. Tannins play a very important role in modifying the protein rheology through non-covalent interactions. Girard et al. (2019) reported that gluten strength in wheat seeds is determined by the cross-linking of condensed tannins, which ultimately enhances the viscosity of the protein. Here, we analyzed the total tannin content in the grains of diverse genotypes of wheat and observed the highest tannin content in wheat cv. HI1544 (70.5 µg/g) and the lowest in wheat cv. HD2329 (21.8 µg/g) (**Figure 5b**). All the popular *cvs*. were observed to have higher tannin content compared to other genotypes. We observed a large positive correlation between the TPC and total tannin content [*r*(16) =0.904, *p*<.001]. Wang et al (2017) observed an increase in the strength of wheat dough due to the presence of hydrolysable tannins. Khazaei and Vandenberg (2020) reported a negative effect of tannin on mineral accumulation and protein content of seeds in Faba beans. It also compromises the bioavailability of various nutrients present in the seeds, influencing the germination process. Ferulic acid (4-hydroxy-3-methoxycinnamic acid) has a very high radical scavenging capacity and is recommended due to its anti-ageing and anticancer properties (Wang et al., 2017). *p*-Coumaric and ferulic acid have been reported to be predominantly accumulated in the wheat bran/pericarp fraction, which shows very high antioxidant potential in grains (Boz, 2015).

### Variations in the polysaccharide accumulation inside the grains

#### Total starch content in the grains

**Figure 6:**
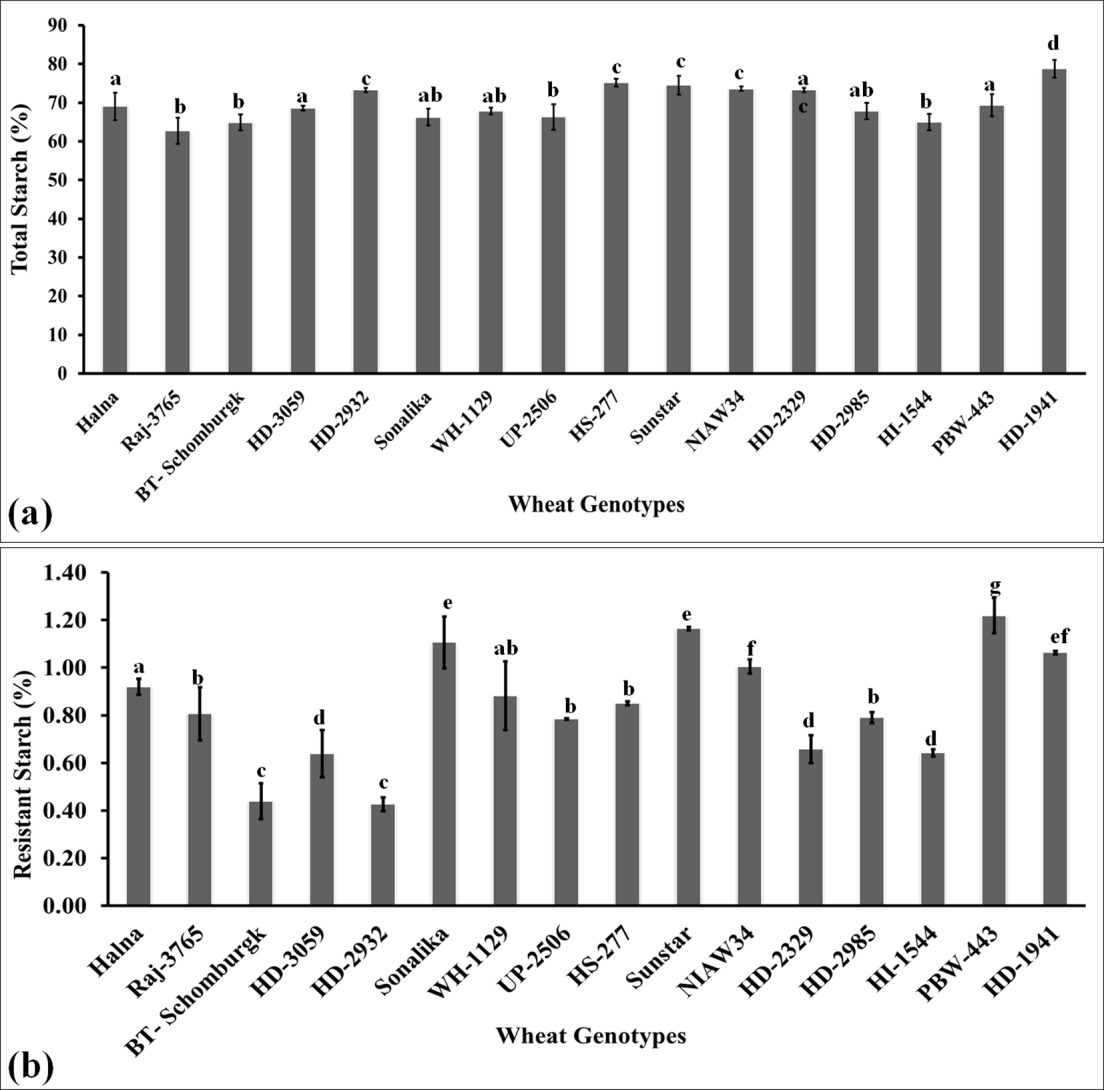
Variations in the polysaccharide (starch and resistant starch) content in the grains of diverse genotypes of wheat. **(a)** Total starch estimation in the grains. **(b)** Resistant starch content in the grains. In total, 16 diverse genotypes of wheat were selected for the study. The vertical line above the bar denotes the *SEM* (n=3).

The total starch was estimated in the grains of diverse genotypes of wheat selected from the core set of wheat developed for their nutritional composition. Starch is a polysaccharide consisting of amylose and amylopectin and is predominant in the grains of cereals. The highest starch level was observed in the grains of wheat cv. HD1941 (79.2%) followed by HS-277 (76.8%) and Sunstar (75.9%) (**Figure 6a**). We observed significant variation in the accumulation of starch in the grains of diverse wheat genotypes. Popular wheat *cvs*., such as Raj3765, HD2329, BT-Schomburgk, and HD2320, also showed significant accumulation of starch in the grains. Wide diversity in the functionality of starch (composition, structure, and granule size) has been observed in different wheat *cvs*. across the world due to variations in genetic and environmental conditions. *Triticum aestivum* or bread wheat is widely grown all over the world with the highest starch content, followed by *T. durum* (Shevkani et al., 2017). Durum wheat is rich in yellow pigment and proteins and is used for making pasta. *T. monococcum*, *T. dicoccum*, and *T.* sp*elta* are ancient species mainly grown in Ethiopia, Turkey, France, and Iran and have little starch, as compared to common wheat or durum wheat.

#### Resistant starch content in the grains

RS is one of the fractions of starch present in the endospermic tissue of the grains and is a slow-digestible starch. It is recommended in the diet of diabetic patients and is considered one of the desirable traits for analyzing the quality of the grains (Khan et al., 2020). We observed the highest RS content in the grains of wheat cv. PBW443 followed by Sunstar and Sonalika (**Figure 6b**). The variations in RS were observed to be significant across the genotypes. The lowest RS content was observed in the wheat *cvs*. HD2932 and BT-Schomburgk. The mechanism underlying the synthesis of RS and significant variations across the genotypes is not known, though pullulanase and starch debranching enzymes have been reported to be associated with the synthesis of RS. RS has been reported to improve the viscoelasticity properties of dough in wheat (Xie et al., 2018). The results of Pearson’s correlation indicated that there was a non-significant small positive relationship between starch and resistant starch, [*r*(16) = 0.286, *p* =0.283]. This is the reason behind the high starch content in the grains of cereals, though the RS content is very low, as compared to millets. Recent studies have identified a positive correlation between starch and RS content in wheat, influenced by factors such as amylose content, starch molecular structure, and processing conditions. Research involving 129 wheat accessions found that RS content varied from 0.07% to 0.47%, with higher amylose content associated with increased RS levels (Liu et al., 2024).

### Effect of thiol on the accumulation of anti-nutritional factors in the grains

#### Phytic acid and total phosphorus content in the grains of diverse wheat genotypes

**Figure 7:**
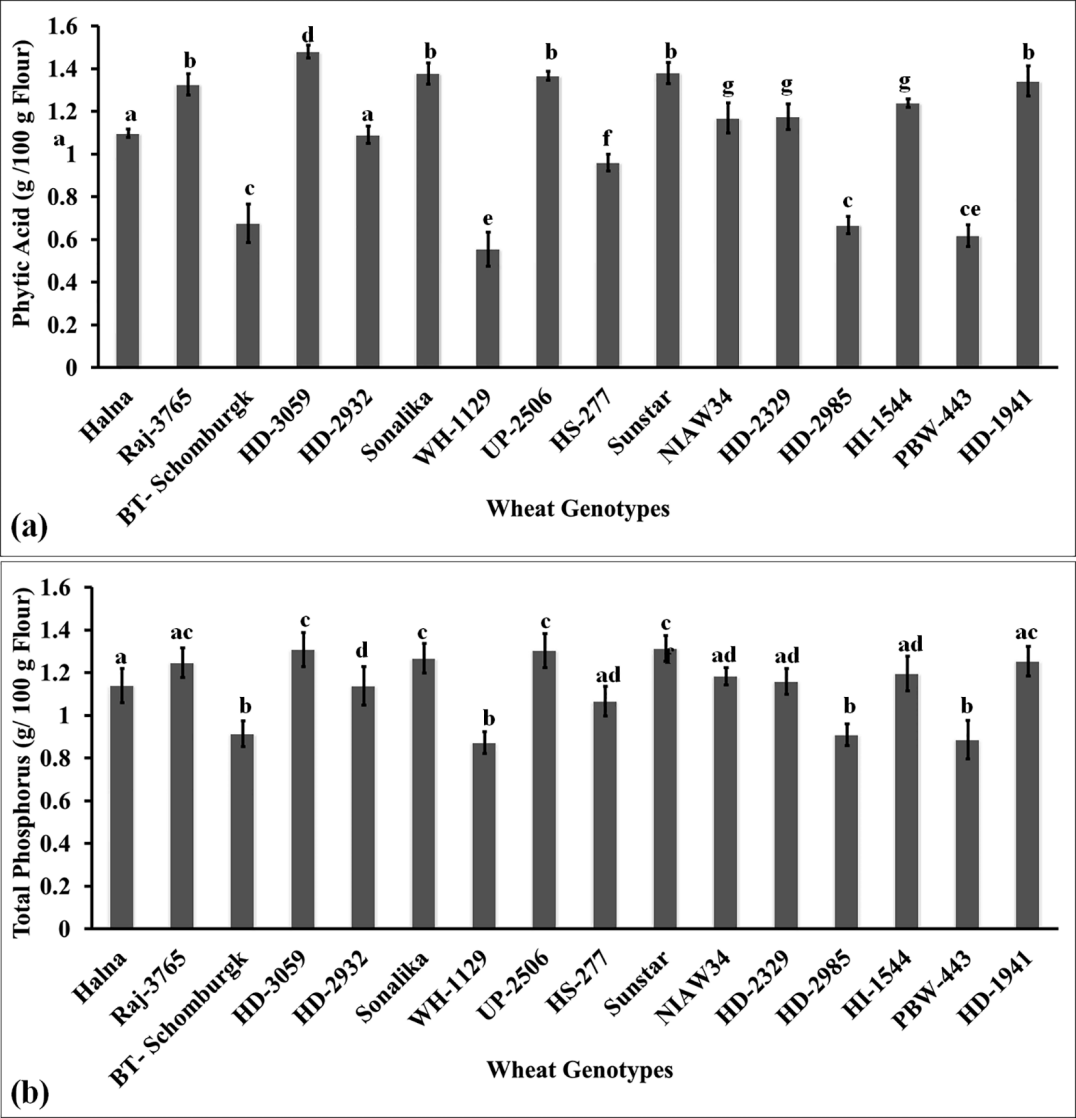
Characterizing the anti-nutritional factor (ANF) present in the grains of diverse genotypes of wheat. **(a)** Phytic acid content in the grains. **(b)** Total phosphorus content in the grains. In total, 16 diverse genotypes of wheat were selected for the study. The vertical line above the bar denotes the *SEM* (n=3); a letter above a bar shows the significance level (P<0.05).

Phytic acid is one of the anti-nutritional factors that compromises the quality of flour. It limits the bioavailability of micronutrients such as iron and zinc and has many effects on the health of humans. We also analyzed the phytic acid content in the grains of diverse genotypes of wheat. The phytic acid content was observed to be the lowest in the grains of wheat cv. WH-1129 (0.56 g/100g) followed by PBW443 (0.62 g/100g) and BT-Schomburgk (0.68 g/100g) (**Figure 7a**). The phytic acid content was observed to be the highest in wheat cv. HD3059 (1.48 g/100g) followed by Sonalika (1.38 g/100g). Most of the *cvs*. selected for the nutritional analysis in the present investigation showed a high content of phytic acid in the grains. A study involving 12 wheat genotypes reported significant variability in phytic acid content, ranging from 0.48% to 1.95%, with an average of 1.24%. This variation in phytic acid levels influenced the nutritional and physical parameters of the wheat grains, highlighting the importance of phytic acid in determining grain quality (Shujaat et al., 2024). The accumulation of high phytic acid has been reported to form complexes with micronutrients such as iron and zinc and restrict their bioavailability inside the body. The results of Pearson’s correlation indicated that there was a non-significant, very small negative relationship between phytic acid and thiol content [*r*(16) = -0.0552, *p* = 0.839]. Phytic acid binds to metal ions and forms stable complexes. Thiol groups present in proteins coordinate with metal ions and maintain the structure and activity of proteins. In contrast to this, phytic acid restricts the availability of metal ions required by the thiol group to maintain the function of proteins in food. The stability and activity of the protein are ultimately compromised. Graminho et al. (2015) reported that the phytase enzyme requires thiol for its activity, acts on the breakdown of phytic acid, and is mainly responsible for the chelation of beneficial micronutrients.

Total phosphorus accumulation in grain is considered one of the desirable traits when analyzing the quality of flour. It has been reported that phosphorus accumulation in grains determines the appearance of grains. It is considered one of the desirable traits for deciding the quality of grains. We observed the highest P accumulation in the grains of wheat cv. HD3059 (1.3 g/100g) followed by UP2506 and Sunstar (**Figure 7b**). Most of the genotypes showed non-significant variations (p<0.05) in the accumulation of phosphorus in grains. The P accumulation was observed to be the lowest in wheat cv. WH1129 (0.872 g/100g). The phosphorus is utilized as a cofactor by different enzymes involved in various metabolic reactions. Lipid composition and fatty acid profile of the seeds were observed to be affected by P deficiency (Xie et al., 2018). Phosphorus in the endospermic tissue has been reported to enhance the synthesis of total storage proteins in the seeds. Phosphorus has been reported to enhance the biosynthesis of starch during the early stages and degradation of starch during the later stages of grain development by triggering the expression of enzymes responsible for starch synthesis and degradation (Zhang et al., 2021). We observed a positive correlation between the starch and the phosphorus contents in flour/meal. Phosphorus enhances the synthesis of starch-bound lipids (phospholipids). Cereals have very little starch-bound phosphate compared to root and tuber crops.

We observed a significantly large positive relationship between phytic acid and total phosphorus [*r*(16) = 0.995, *p*< 0.001] and a negative correlation with thiol [*r*(16) = -0.20, *p*< 0.001].

#### Variations in the total protein and gluten content in grains

**Figure 8:**
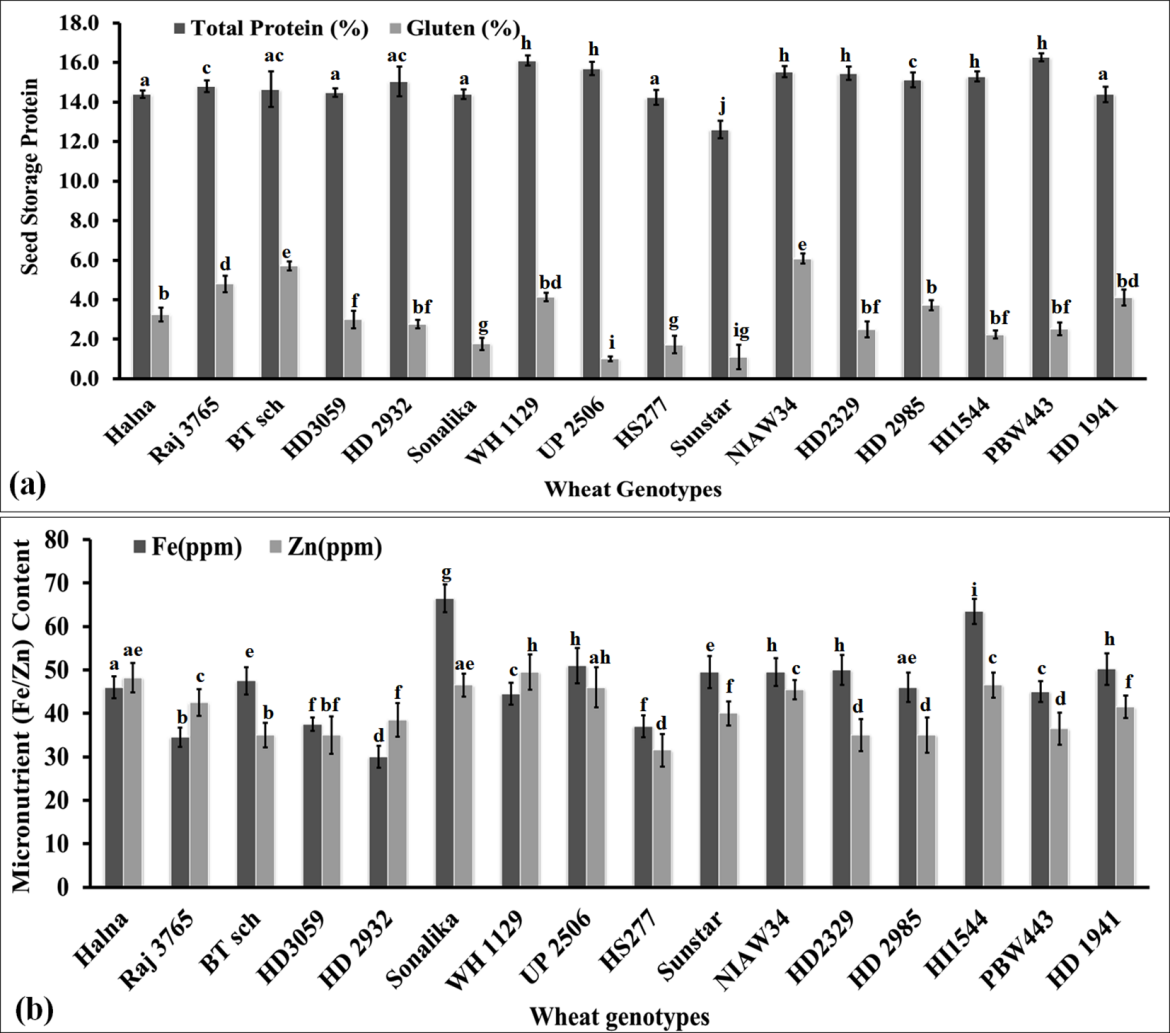
Variations in the seed storage proteins (SSPs) and micronutrient content in the grains of diverse genotypes of wheat. **(a)** Total seed storage proteins in the grains. **(b)** Iron and zinc content in the grains. In total, 16 diverse genotypes of wheat were selected for the study. The vertical line above the bar denotes the *SEM* (n=3).

We analyzed the variations in the seed storage protein in terms of total protein and gluten content in the grains of diverse genotypes of wheat. The total protein content was observed to be the highest (16.1%) in wheat cv. PBW443 followed by WH1129 (15.8%), whereas it was observed to be the lowest (12.8%) in cv. Sunstar (**Figure 8a**). Most of the *cvs*. showed a non-significant difference in their total protein content. Pearson’s correlation coefficient showed a negative correlation between the total protein and thiol content [*r*(16) = -0.25, *p*< 0.001]. Hussein and Alshammari (2022) reported that cysteine modulates amino nitrogen, total phenols, and the synthesis of new proteins in plants under adverse conditions.

Furthermore, we estimated the gluten content in the grains of diverse genotypes of wheat. The highest gluten content (6.2%) was observed in wheat cv. NIAW34 followed by BT-Schomburgk (5.9%), whereas the lowest (0.9%) gluten content was observed in cv. UP2506 (**Figure 8a**). We observed significant (p<0.05) differences in the gluten content among the diverse genotypes. However, we could not establish any correlation between the total protein and gluten content in the grains. The gluten content was observed to be the highest in thermosusceptible wheat *cvs*. with a low Cys/Met ratio. A non-significant negative correlation was established between the thiol and gluten contents in the wheat grains. We observed that the *cvs*. that are popular among consumers had a total protein content >14% and gluten content above 2%. Veraverbeke and Delcour (2002) observed a linear correlation between the total protein and gluten content in wheat grains. Tea et al. (2005) observed that the abundance of S-containing amino acids in the grains modifies the degree of polymerization of storage proteins and influences the rheological properties of dough.

#### Variations in the micronutrient content in diverse genotypes of wheat

We analyzed the Fe and Zn contents in the grains of diverse genotypes of wheat. Iron content was observed to be the highest (68.0 ppm) in wheat cv. Sonalika, followed by HI1544 (65.0 ppm), whereas Zn was observed to be the highest (52 ppm) in cv. WH1129 (**Figure 8b**). We observed significant variations (p<0.05) in the Fe and Zn content in the grains of diverse genotypes of wheat. Most of the sensitive *cvs*., such as BT-Schomburgk, HD3059, HS277, and NIAW34, were observed to have high Fe and Zn contents compared to the tolerant *cvs*., such as Halna, HD2985, and HD2932. Pearson’s correlation coefficient showed a positive correlation between the Fe/Zn content and thiol content [*r*(16) = 0.15, *p*< 0.023; 0.35, *p*< 0.001]. The Fe/Zn variations among the genotypes were an inherited characteristic of the cv., which changes drastically under stress or adverse environmental conditions.

Based on the present findings and the literature available in the public domain, we modeled a simple pathway of activity of a thiol-based redox sensor. ROS produced inside the cells in response to different environmental and internal conditions act on active thiols and oxidize the catalytic Cys group and form inter- or intramolecular S–S–, –SOH, –SO_2_H, and –SO_3_H, which modify the structure and interactions of different proteins and enzymes involved in various metabolic pathways and ultimately regulate the accumulation of various metabolites inside the cells.

**Figure 9:**
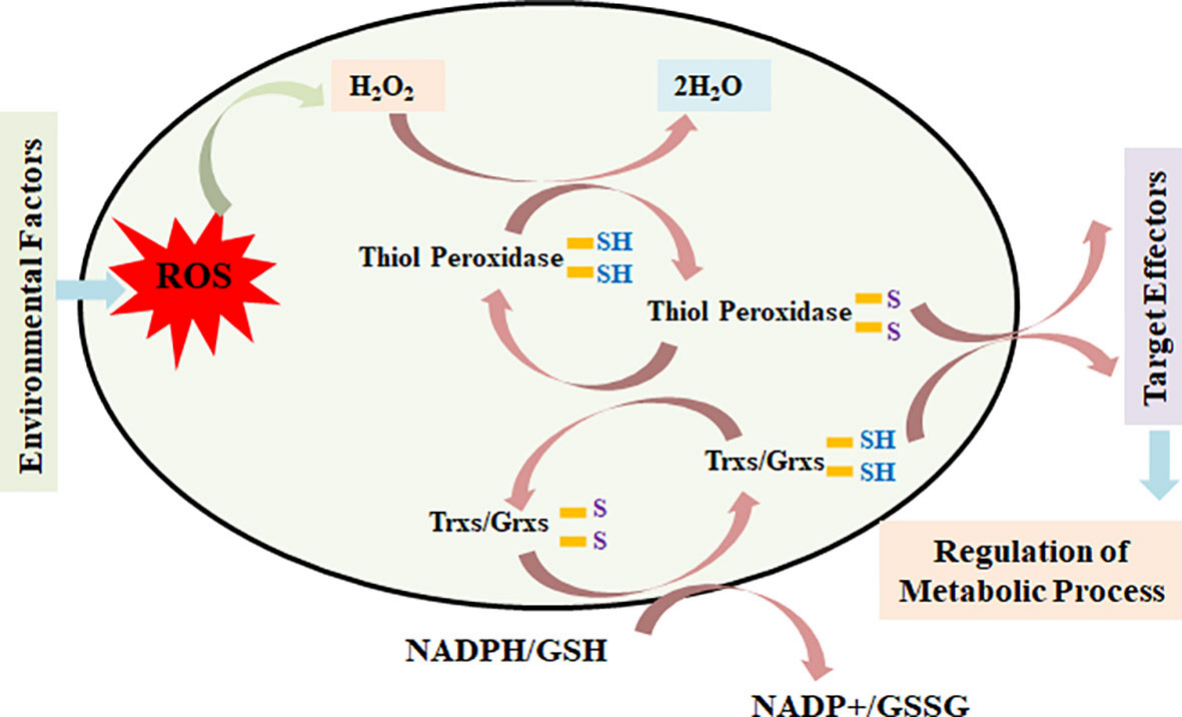
Model depicting the role of thiol-based redox sensors—thiol peroxidase, thioredoxin, glutaredoxin, and glutathione reductase—in transmitting the redox signal to effector target genes/proteins and, in turn, modulating the expression/activities of key genes/proteins linked with different metabolic pathways.

ROS also acts on thiol peroxidases such as TPXs and GPXs and oxidizes the Cys residue into different forms and transfers their redox signal to target genes and proteins linked to different metabolic processes (**Figure 9**). Similarly, Trx and Grx proteins are oxidized at the Cys center by thiol peroxidase and simultaneously are reduced by electrons received from NADPH/GSH with the help of thiol reductases. Trx/Grx proteins play the role of redox sensors through their conversion from the oxidized to reduced form and *vice versa* in response to different cellular and environmental conditions. This regulates the target effectors and, in turn, modulates the different metabolic processes operating inside the plants.

Thiol-based redox sensors can be used in wheat breeding programs to improve the stress tolerance and grain quality of the wheat and regulate antioxidant pathways, and can be integrated with advanced breeding techniques such as CRISPR and marker-assisted selection to accelerate the development of wheat varieties with desired traits.

## Conclusions

Thiol-based redox sensors are essential in regulating various processes such as defense, growth, and the accumulation of metabolites within plant tissues. To explore their role, we characterized 16 diverse wheat genotypes and observed significant variations in thiol and disulfide contents in the grains. Wheat cultivar Halna exhibited the highest thiol content, followed by HD2985 and Raj3765. This pattern was confirmed through expression analysis of genes involved in thiol-based redox sensing, such as TRX, GRX, and GR, in the developing endosperm. The highest relative fold expression of these genes was observed in Halna, considered a tolerant cultivar, during the seed hardening stage of endosperm development, compared to sensitive cultivars. Amino acid analysis of 11 biosynthetic genes showed that GBSS, involved in starch biosynthesis, had the highest Cys content (2.25%), while BCH, involved in tannin synthesis, had the highest Met content (4.04%). The Cys: Met ratio was >1.5 in tolerant genotypes such as HD2985, Halna, and HD2932, whereas it was<1.5 in sensitive cultivars such as BT-Schomburgk and PBW343. Wheat cv. NIAW34, with a Cys: Met ratio >2.1, had the highest yellow pigment content (6.08 µg/g dry matter). Genotypes with higher Cys: Met ratios also had higher total TPC and tannins. A positive correlation was observed between polysaccharide (starch and resistant starch) accumulation and thiol content. This approach could contribute to the development of wheat with improved proximate compositions, addressing the challenges posed by global climate change and ensuring “Food for the Future.”

## Data Availability

The original contributions presented in the study are included in the article/**Supplementary Material.** Further inquiries can be directed to the corresponding author.
