# Supplementary material for: Thiol-based redox sensing regulates the yellow pigment and antioxidant accumulation and improves the nutritional quality of wheat grains (Triticum aestivum L.)
Source: Front Plant Sci. 2025 May 30;16:1488697. doi: 10.3389/fpls.2025.1488697 (PMC12162979; doi:10.3389/fpls.2025.1488697)
Supplement: Supplementary file 1 [file Table1.docx]

**Table S1.** List of wheat genotypes used for the characterization of thiol-based sensing activity and effect on the nutritional composition and grain quality.

| **Genotypes** | **Nature of cultivar** |
| --- | --- |
| Halna | Tolerant |
| Raj3765 | Tolerant |
| BT-Schomburgk | Susceptible |
| HD3059 | Tolerant |
| HD2932 | Tolerant |
| Sonalika | Susceptible |
| WH1129 | Susceptible |
| UP2506 | Tolerant |
| HS277 | Susceptible |
| Sunstar | Susceptible |
| NIAW34 | Susceptible |
| HD2329 | Susceptible |
| HD2985 | Tolerant |
| HI1544 | Tolerant |
| PBW343 | Susceptible |
| HD1914 | Susceptible |
